# Mapping Colorectal Practice Patterns in a Community Hospital: A Correspondence Analysis Approach

**DOI:** 10.7759/cureus.116650

**Published:** 2026-09-21

**Authors:** Timothy W Gichuru, Abid Qureshi, Luca Milone

**Affiliations:** 1 Undergraduate Medical Education, Brooklyn Hospital Center, New York, USA; 2 School of Graduate Studies, St George's University, New York, USA; 3 General and Colorectal Surgery, Brooklyn Hospital Center, New York, USA

**Keywords:** colorectal, correspondence analysis, laparoscopic, open, robotic surgical procedures

## Abstract

Background: This single-surgeon, single-institution study aims to show the association between perioperative variables and surgical technique in colorectal surgery, assess discrepancies in patient management relative to established guidelines, and identify underlying causes for improvement. Because the findings reflect one surgeon's practice at one hospital, they are intended as a reproducible audit methodology for single providers rather than as generalizable clinical guidance.

Methods: A total of 171 patients underwent colorectal surgery at The Brooklyn Hospital between 2017 and 2024, including 73 robot-assisted, 62 open, and 36 laparoscopic procedures. The primary analysis examined the relationship between surgical technique and diagnosis. Secondary, exploratory analyses evaluated associations between surgical technique and 14 preoperative variables, and surgical technique and 14 postoperative variables. We assessed associations using chi-square tests, with likelihood-ratio chi-square tests when more than 20% of expected cell counts were below 5, along with correspondence analysis. To account for the five planned comparisons, we set statistical significance at α =0.01 using a Bonferroni correction.

Results: The most common surgical indications were ascending colon cancer (27 cases), diverticulitis (25 cases), and colostomy reversal (20 cases). The technique-diagnosis association was statistically significant (χ² = 102.83, df = 28, p < 0.0001, Cramér's V = 0.51): laparoscopic surgery was most frequently associated with volvulus surgery, robotic technique with ascending colon cancer and colovesicular fistula surgeries, and open surgery with ileostomy reversal surgery. The technique-preoperative association was not statistically significant (χ² = 32.71, df = 26, p = 0.17); the exploratory associations included laparoscopic technique with male sex and NSAID use, and robotic technique with elective procedures and prior radiation. The association between surgical technique and postoperative outcomes was significant on likelihood-ratio testing (G = 41.38, df = 26, p = 0.028), but did not meet the Bonferroni-adjusted significance threshold. Correspondence analysis showed that laparoscopic surgery was associated with surgical site infection, while robotic surgery was associated with shorter hospital stays and earlier return of bowel function.

Conclusion: This study explores one surgeon’s practice in the key relationships between surgical techniques and patient characteristics, highlighting their impact on surgical outcomes. It identifies distinct trends in using laparoscopic, robotic, and open surgeries. This practice follows established practice, utilizing preoperative collaboration in optimizing surgical care. The methodology of this analysis, although pertaining to one practice, can be applied to any surgeon’s data to audit practice patterns and identify areas of improvement.

## Introduction

In the modern era of surgery, breakthroughs in advancements in diagnostic technologies, pharmaceutical developments, and innovative surgical technologies seek to improve the quality of care patients receive. However, to truly enhance patient outcomes, astute physicians and researchers must first identify the existing issues in care.

Managing colorectal diseases spans millennia, from the Ancient Egyptians to the modern practice by Board-Certified Colorectal Surgeons and General Surgeons [1]. This practice encompasses the management of various conditions, including colorectal cancer, inflammatory bowel disease (IBD), diverticulitis, bowel obstruction, and proctological disorders [1]. These conditions are managed with various surgical techniques, including open and minimally invasive surgeries [2]. However, colorectal surgery carries potential complications that may alter a patient's postoperative course. The nature and severity of these complications vary and are often influenced by the underlying disease process, preoperative conditions, and the complexity of the postoperative period.

The lack of consensus and existing evidence identifying risk factors for complications in colorectal surgery suggests that physicians may not be aware of how to avoid complications preemptively [3]. Enhanced Recovery Protocol (ERP), a set of standardized perioperative procedures and practices, has been the current standard for those undergoing elective surgery [4]. Advancements in expanding this protocol to urgent or emergent cases, as seen in the Enhanced Recovery After Surgery (ERAS) protocol, have shown improved patient outcomes [5].

The study investigates the methodology to analyze the association of perioperative variables in one surgeon’s practice to determine any underlying issues according to standard guidelines and why discrepancies may exist. Furthermore, the aim of the study is to provide a methodology to uncover relationships in a surgeon’s practice that are unique to their practice, demonstrating the benefits it can provide by enhancing an understanding of practice dynamics and pinpointing areas for improvement.

## Materials and methods

Study design and setting

This retrospective observational study was conducted at The Brooklyn Hospital Center, Brooklyn, NY, USA. The study included all eligible patients who underwent colorectal surgical procedures at the Department of Surgery between January 2017 and December 2024. The objective of the study was to characterize colorectal surgical practice patterns by examining relationships among patient diagnoses, operative approaches, preoperative characteristics, and postoperative outcomes using correspondence analysis together with conventional statistical methods.

Study population and sampling

A retrospective consecutive sampling strategy was employed. All patients who underwent colorectal surgery during the study period and met the eligibility criteria were included. Data were extracted from the hospital-specific electronic medical record (EMR).

Inclusion Criteria

Inclusion criteria were patients who underwent a colorectal surgical procedure between 2017 and 2024, and whose complete operative records and complete preoperative and postoperative clinical information were available in the EMR.

Exclusion Criteria

Exclusion criteria were incomplete medical records, missing critical operative or postoperative variables, and missing operative reports that prevented complete data extraction.

Data collection

Clinical data were collected retrospectively from the institutional EMR using standardized data abstraction procedures. At the time of data collection, all patient information was entered into a Microsoft Excel (Microsoft, Redmond, WA, USA) database and de-identified by assigning each patient a unique alphanumeric identification code. Direct patient identifiers were removed prior to statistical analysis, and only de-identified data were used throughout the study to ensure patient confidentiality.

One hundred and seventy-one patients were categorized by primary preoperative diagnosis, operative approach (open, laparoscopic, or robot-assisted), and postoperative outcomes. A total of 15 diagnostic categories were identified: Rectal Cancer, Sigmoid Cancer, Ascending Colon Cancer, Descending Colon Cancer, Transverse Colon Cancer, Appendiceal Cancer, Peritoneal or Gynecological Cancer, Colovesicular Fistula, Colovaginal Fistula, Diverticulitis, Diverticulitis with Complication, Volvulus, Ileostomy or Colostomy Creation, Colostomy Reversal, and Ileostomy Reversal.

Preoperative variables

The following preoperative variables were collected: age, sex, body mass index (BMI), surgical urgency (elective versus emergency), medical comorbidities, medications for chronic medical conditions, indication for cancer resection, history of previous abdominal surgery, smoking status, alcohol use, anticoagulant therapy, preoperative bowel preparation, receipt of chemotherapy, and receipt of radiation therapy.

Postoperative variables

The postoperative variables included operative time, length of hospital stay (categorized as <6 days or ≥6 days, based on the cohort mean of six days), time to return of bowel function (categorized as <5 days or ≥5 days), estimated blood loss (>500 mL), intraoperative blood product transfusion, vasopressor use, stoma creation, surgical re-exploration during the index hospitalization, intensive care unit admission, surgical site infection, intra-abdominal infection, anastomotic leak, pneumonia, sepsis, and in-hospital mortality.

Outcome measures

The primary aim of the study was to identify associations among operative approach, disease category, and perioperative outcomes using correspondence analysis. Secondary outcomes included identifying relationships between preoperative patient characteristics and operative technique, as well as associations between operative technique and postoperative complications.

Confounding variables

Potential confounding variables considered during the analysis included patient age, sex, BMI, comorbidity burden, history of previous abdominal surgery, elective versus emergency surgery, neoadjuvant chemotherapy, neoadjuvant radiation therapy, smoking status, alcohol use, and anticoagulant therapy. These variables were included in the exploratory analyses because they may influence both surgical approach and postoperative outcomes.

Statistical analysis

Statistical analyses were performed using IBM SPSS Statistics Version 29.0.2.0 (IBM Corp., Armonk, NY, USA). Data were initially reviewed for completeness, consistency, duplicate entries, implausible values, and missing information before analysis. Patients with incomplete records or missing critical variables were excluded according to the predefined exclusion criteria. No imputation of missing data was performed. Descriptive statistics were generated for all study variables. Categorical variables are presented as frequencies and percentages. Cross-tabulation analyses were performed to summarize the distribution of preoperative characteristics by diagnosis and postoperative outcomes by operative technique. Associations between categorical variables were evaluated using Pearson's chi-square (χ²) test of independence. Expected cell frequencies were assessed to verify the assumptions of the chi-square test. When more than 20% of expected cell counts were fewer than 5, Fisher's exact test or the likelihood-ratio chi-square statistic was used, as appropriate. To account for multiple hypothesis testing, Bonferroni correction was applied where appropriate to reduce the probability of Type I error arising from multiple simultaneous comparisons. Effect sizes for statistically significant associations were quantified using Cramér's V. Standardized residuals were examined in post hoc analyses to identify the cells contributing most strongly to the associations, with standardized residuals greater than ±2 considered indicative of meaningful departures from expected frequencies.

Correspondence analysis was subsequently performed to explore and visualize multidimensional relationships among categorical variables. Correspondence analysis is an established multivariate exploratory technique for analyzing contingency tables that graphically represents associations between categories by projecting them into a low-dimensional space while preserving the underlying chi-square distances, thereby facilitating the interpretation of complex categorical data [6,7]. This approach enables the identification and visualization of relationships among diagnoses, operative techniques, preoperative characteristics, and postoperative outcomes that may not be readily apparent using conventional univariate analyses [6]. The interpretation of correspondence maps, including the relative proximity of category points and their contribution to the principal dimensions, was performed according to established methodological recommendations described by Greenacre [7] and Higgs [8]. The methodology implemented in this study therefore follows previously validated statistical approaches for exploratory analysis of categorical clinical data [6-8]. All statistical tests were two-sided, and statistical significance was defined as a p-value <0.05.

Sample size

Because this was a retrospective observational study, no formal a priori sample size calculation was performed. All consecutive patients meeting the eligibility criteria during the study period were included to maximize statistical power and minimize selection bias.

Ethical considerations

The study was conducted in accordance with institutional ethical standards. Patient information was obtained retrospectively from the institutional EMR. All study data were de-identified before statistical analysis by assigning unique alphanumeric study codes to preserve patient confidentiality. Institutional Review Board approval was obtained prior to the commencement of the study, and the requirement for informed consent was waived owing to the study's retrospective nature and the use of de-identified clinical data.

## Results

The cohort comprised 171 patients. Surgical techniques and diagnoses split into patient groups were 73 robot-assisted cases, 62 open cases, and 36 laparoscopic cases (Figure 1, Table 1). The distribution of preoperative variables across the study population was diverse, reflecting the cohort's clinical heterogeneity as seen in Table 2. Additionally, Figure 2 illustrates the postoperative variables (complications) associated with specific surgical techniques. The indications for surgery with total patient numbers are outlined in Table 1. The most common indication for operation was ascending colon cancer in 27 patients, followed by 25 patients for diverticulitis. Colostomy reversal was recorded in 20 cases, while rectal cancer was observed in 19 cases. There were 16 cases of ileostomy reversal and 15 cases of sigmoid cancer. Colostomy creation and colovesicular fistula each had nine cases. Diverticulitis with complications was reported in six cases. Both transverse colon cancer and volvulus had five cases each. Appendiceal cancer, descending colon cancer, and colovaginal fistula were each observed in four cases. The least common condition in this dataset was peritoneal or gynecological cancer, with three cases.

**Figure 1:**
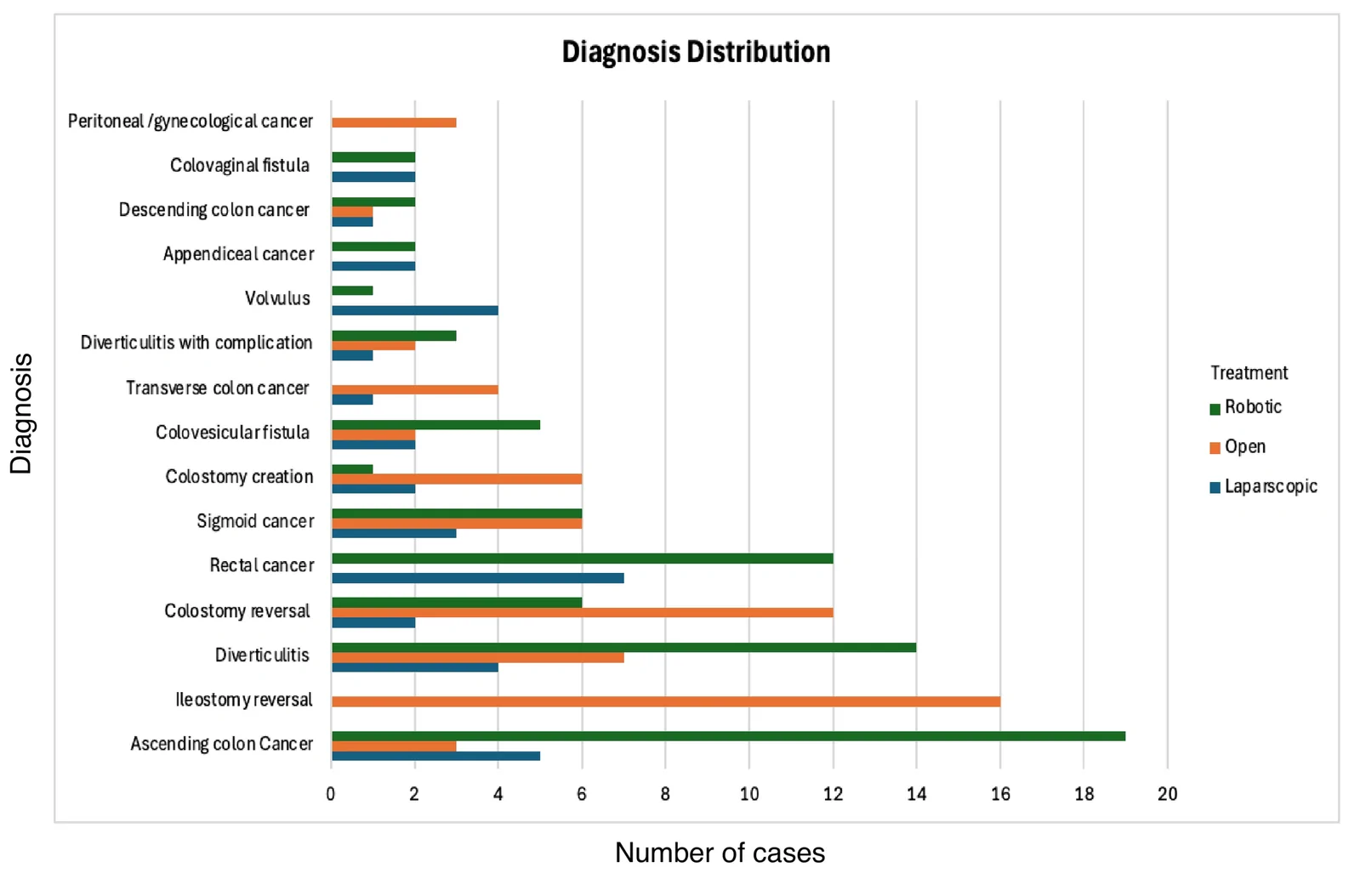
The distribution of diagnoses per surgical technique.

**Table 1:** Total number of patients with indications for surgery by diagnosis

Diagnosis	No. of patients
Ascending colon cancer	27
Diverticulitis	25
Colostomy reversal	20
Rectal cancer	19
Ileostomy reversal	16
Sigmoid cancer	15
Colostomy creation	9
Colovesicular fistula	9
Diverticulitis with complication	6
Transverse colon cancer	5
Volvulus	5
Appendiceal cancer	4
Descending colon cancer	4
Colovaginal fistula	4
Peritoneal or gynecological cancer	3
Total	171

**Table 2:** Preoperative variables assessed in this study and their frequencies

Preoperative variable	n (of 171)	%
Comorbidities present	138	80.7
Bowel preparation performed	144	84.2
Elective surgery	125	73.1
Comorbidities requiring chronic medication	125	73.1
Prior abdominal surgery	107	62.6
Age >65 years	96	56.1
Male sex	71	41.5
Cancer resection performed	71	41.5
NSAID use	59	34.5
BMI-based indicator	119	69.6
Current smoker	29	17.0
Chemotherapy prior to surgery	29	17.0
Alcohol use	28	16.4
Radiation prior to surgery	20	11.7

**Figure 2:**
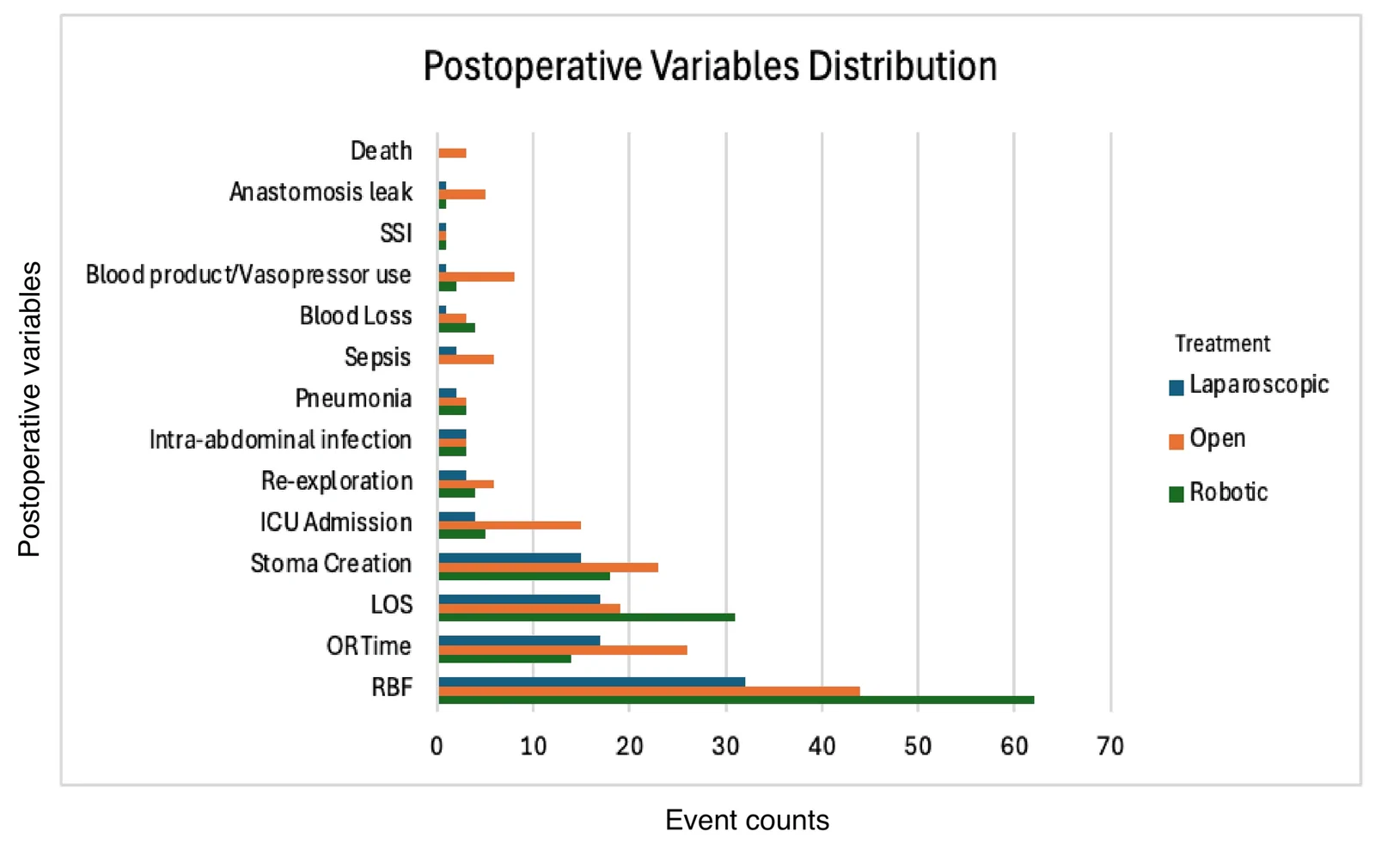
The distribution of postoperative variables per surgical technique. Listed postoperative variables on y-axis: Death, anastomosis leak, SSI, blood product/vasopressor use, blood loss, sepsis, pneumonia, intra-abdominal infection, re-explorations, ICU admission, stoma creation, LOS, OR, and RBF. SSI, surgical site infection; LOS, length of stay; OR, operative time; RBF, return-of-bowel function.

Correspondence analysis

Within the dataset, an analysis was performed to identify the relationship between the distinct surgical techniques and preoperative and postoperative variables, including 14 variables for each group. Additionally, the study explored how different diagnoses correlated with the incidence of these preoperative and postoperative variables and assessed the strength of these relationships. To do this, a correspondence analysis was selected to allow for simultaneous assessment of the relationships between multiple categorical variables [6]. Correspondence analysis provides a view of the relationships among all variables, and this is done through dimension reduction and visualization on a plot graph [6,7].

When performing a correspondence analysis, comparing one set of differing variables against another set of variables results in individualized axes, which can be reduced and plotted onto a single graph. This graph's axes describe dimensions constructed by capturing the most significant source of variation, while the second dimension captures the most important pattern. Each additional dimension captures smaller, more specific patterns. Together, these dimensions help reveal how categories are related to one another in a simplified, visual way. This process is called "dimension reduction" [6,8]. Doing so allows us to visualize the closeness and clustering of points, enabling interpretation of correspondence and identifying existing relationships within the distribution of the dataset. The output plot and coordinates of those points are crucial for describing the strength of these relationships [8]. As proximity can indicate a relationship, the strength of that relationship is important because it helps quantify the significance and relevance of the association between the variables. Stronger relationships are more likely to have practical implications. The angles are calculated based on the angle between the vectors originating from the origin (0,0) and their respective coordinates. These angles can vary in magnitude and direction. The smaller the angle, the stronger the relationship, and the direction, which is either positive or negative, indicates association and contra-association, respectively.

Surgical technique and diagnosis

The association between surgical technique and diagnosis was statistically significant. Because 68.9% of expected cell counts were below 5, the likelihood-ratio chi-square test was reported: G = 102.83, df = 28, p < 0.0001 (Pearson χ² = 88.21, p < 0.0001, concordant). Cramér's V = 0.51, indicating a large effect. This remains significant after Bonferroni correction (α = 0.01) (Table 3). Figure 1 shows the relative frequency of technique by diagnosis; for example, of 19 rectal cancer cases, seven were laparoscopic, 12 robotic, and none open.

**Table 3:** Chi-square test statistics for correspondence analyses of surgical technique Pearson's chi-square (χ²) or likelihood-ratio chi-square (G) test results for the association between surgical technique and diagnosis, preoperative variables, and postoperative variables. The likelihood-ratio statistic was used in place of Pearson's χ² wherever more than 20% of cells had an expected count <5, per the prespecified statistical plan. Significance is reported at both the conventional threshold (α = 0.05) and after Bonferroni correction for the five planned comparisons (α = 0.01). *N for the preoperative and postoperative analyses reflects total indicator-cell counts across the 14 binary variables per patient, not patient count, as each patient contributes to multiple variable cells; the underlying cohort remains n = 171 throughout.

Relationship	N	Table size	Test used	Statistic	df	p-Value	Cramér's V	Cells with expected <5	Conclusion (α = 0.01)
Technique × Diagnosis	171	3 × 15	χ² (LR/G)	102.83	28	<0.0001	0.508	68.9%	Significant
Technique × Preoperative variables	1101*	3 × 14	χ² (Pearson)	32.71	26	0.171	0.122	2.4%	Not significant
Technique × Postoperative variables	412*	3 × 14	χ² (LR/G)	41.38	26	0.028	0.215	61.9%	Significant (uncorrected); nonsignificant after Bonferroni

Standardized (adjusted) residual analysis identified the following cells as the strongest departures from independence (|adjusted residual| ≥ 2): open surgery for ileostomy reversal (observed 16 vs. expected 5.8; residual = 5.6); absence of open surgery for rectal cancer (observed 0 vs. expected 6.9; residual = −3.5); laparoscopic surgery for volvulus (observed 4 vs. expected 1.1; residual = 3.3); robotic surgery for ascending colon cancer (observed 19 vs. expected 11.5; residual = 3.2); underuse of open surgery for ascending colon cancer (observed 3 vs. expected 9.8; residual = −3.0); open surgery for colostomy reversal (observed 12 vs. expected 7.3; residual = 2.4); open surgery for peritoneal/gynecological cancer (observed 3 vs. expected 1.1; residual = 2.3); absence of laparoscopic surgery for ileostomy reversal (observed 0 vs. expected 3.4; residual = −2.2); and open surgery for transverse colon cancer (observed 4 vs. expected 1.8; residual = 2.1).

The correspondence map (Figure 3) captures 100% of total inertia in its two plotted dimensions (Dimension 1 = 83.3%, Dimension 2 = 16.7%) (Table 4); because the technique variable has only three categories, no association is lost by reducing to two dimensions. Laparoscopic surgery clustered with volvulus, appendiceal cancer, and colovaginal fistula; robotic surgery with ascending colon cancer, colovesicular fistula, diverticulitis, and diverticulitis with complication; and open surgery with sigmoid cancer, colostomy reversal, ileostomy reversal, and peritoneal/gynecological cancer. Rectal cancer and descending colon cancer showed a clear contra-association with open surgery, and transverse colon cancer and colostomy creation showed a contra-association with robotic surgery.

**Figure 3:**
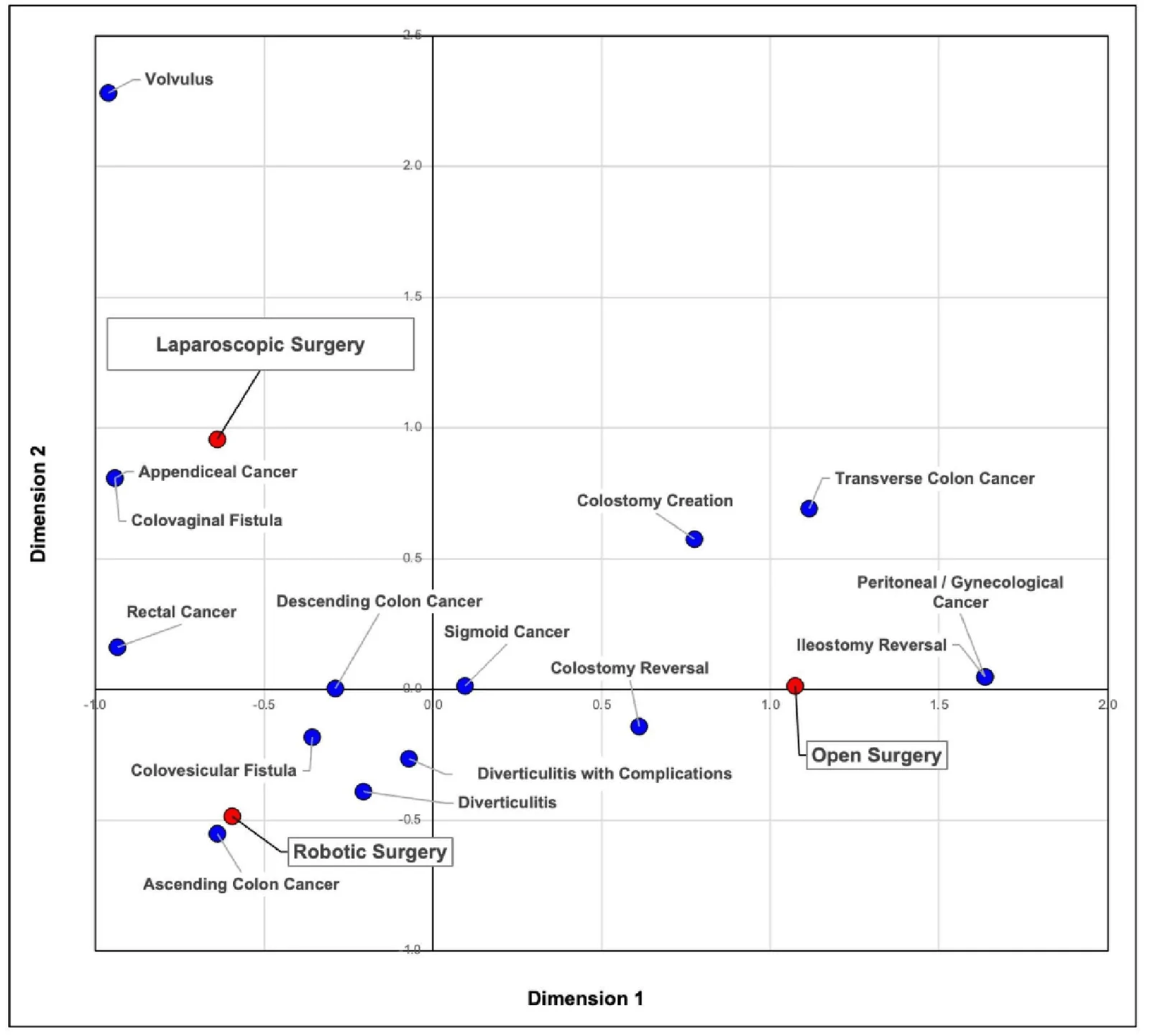
Correspondence graph examining surgical technique (Tx) and diagnosis (Dx). Dimension 1 and Dimension 2 represent the first two latent correspondence analysis dimensions that account for the greatest variation in the association between diagnoses and operative approaches. Categories positioned closer together demonstrate stronger associations, whereas those located farther apart or in opposite directions indicate weaker or contra-associations. Observations and interpretation: Laparoscopic surgery clustered with volvulus, appendiceal cancer, and colovaginal fistula, suggesting these diagnoses were managed more frequently using minimally invasive laparoscopy. Robotic surgery was associated with ascending colon cancer, colovesical fistula, diverticulitis, and diverticulitis with complications. Open surgery clustered with sigmoid cancer, colostomy reversal, ileostomy reversal, and peritoneal/gynecological cancer, indicating these diagnoses were more commonly managed through an open approach. Rectal cancer and descending colon cancer demonstrated a clear contra-association with open surgery, whereas transverse colon cancer and colostomy creation were contra-associated with robotic surgery.

**Table 4:** Percentage of total inertia captured by correspondence analysis dimensions Percentage of total inertia (chi-square distance) accounted for by Dimension 1 and Dimension 2 of each correspondence analysis map (Figures 3, 4, 5), obtained by singular value decomposition of the standardized residual matrix. Because the technique variable has only three categories, the two plotted dimensions capture 100% of the total association for all three technique-based analyses, confirming that no structure is lost in the two-dimensional visualization.

Relationship	Dim. 1 inertia (%)	Dim. 2 inertia (%)	Cumulative (Dims 1 + 2) (%)
Technique × Diagnosis (Figure 3)	83.3	16.7	100.0
Technique × Preoperative (Figure 4)	71.1	28.9	100.0
Technique × Postoperative (Figure 5)	86.4	13.6	100.0

**Figure 4:**
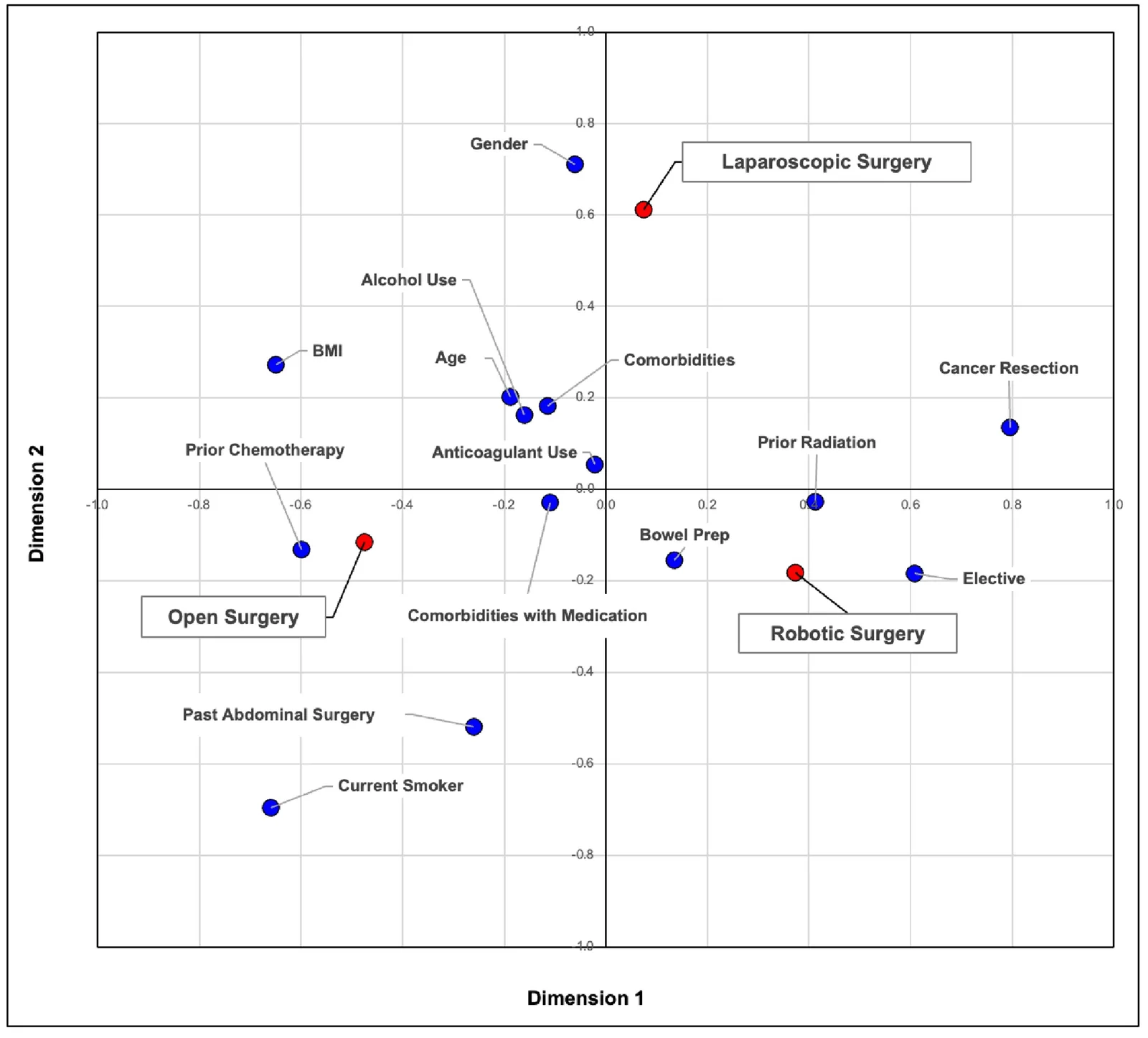
Correspondence graph examining surgical technique (Tx) and preoperative variables. Dimension 1 and Dimension 2 represent the first two latent correspondence analysis dimensions describing the relationships between operative approach and preoperative patient characteristics. Variables positioned closer to a surgical technique indicate stronger associations, whereas variables located in opposite directions indicate contra-associations. Observations and interpretation: Laparoscopic surgery was associated with male sex and anticoagulant use. Robotic surgery clustered with elective procedures, bowel preparation, prior radiation therapy, and cancer resections, reflecting greater use in planned oncologic operations. Open surgery was associated with prior chemotherapy and patients requiring medications for comorbid conditions. BMI <25, alcohol use, and younger age showed a contra-association with robotic surgery, while elective procedures, prior radiation, and cancer resections demonstrated a contra-association with open surgery. Prior abdominal surgery was infrequently associated with laparoscopic procedures.

**Figure 5:**
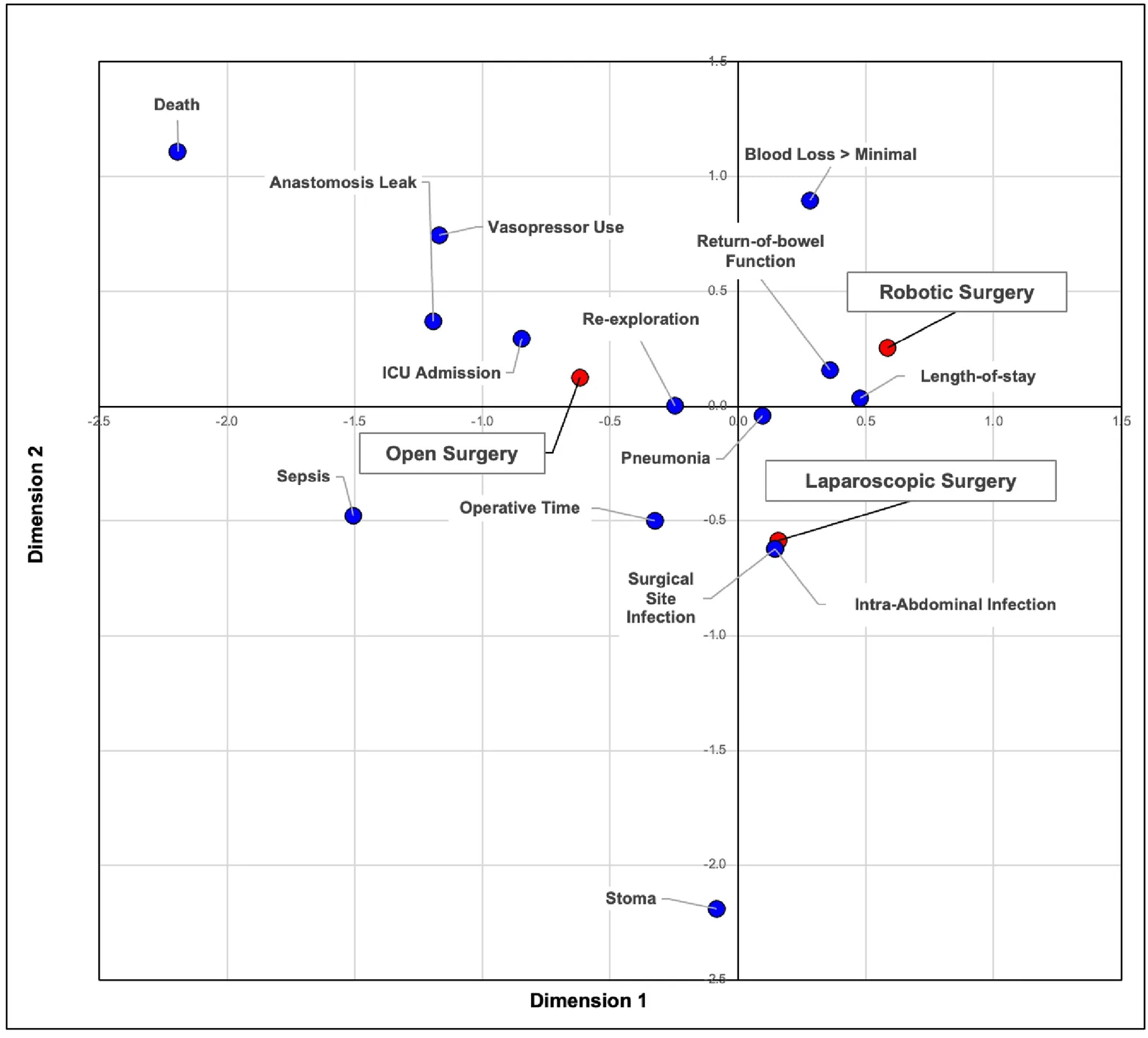
Correspondence graph examining surgical technique (Tx) and postoperative variables. Dimension 1 and Dimension 2 represent the first two latent correspondence analysis dimensions summarizing the associations between operative approach and postoperative outcomes. Variables located near a surgical technique are more strongly associated with that approach, whereas greater separation indicates weaker or negative associations. Observations and interpretation: Open surgery clustered with ICU admission, vasopressor use, re-exploration, sepsis, anastomotic leak, and in-hospital mortality, reflecting greater association with more complex postoperative courses. Robotic surgery was associated with earlier return of bowel function, minimal blood loss, and shorter hospital length of stay. Laparoscopic surgery clustered with surgical site infection and intra-abdominal infection but demonstrated no clear contra-associations with postoperative variables. In contrast, robotic surgery showed contra-associations with re-exploration and sepsis, while open surgery demonstrated contra-associations with shorter hospital stay and pneumonia.

Surgical technique and preoperative variables

This association was not statistically significant: χ² = 32.71, df = 26, p = 0.17 (only 2.4% of expected cells were <5, so the Pearson statistic is appropriate here). Cramér's V = 0.12, a small effect (Table 3). Because the global test is not significant, the following correspondence-map associations (Figure 4) are reported as exploratory/hypothesis-generating rather than confirmed: laparoscopic surgery clustered with male sex and NSAID use; robotic surgery clustered with elective procedures, bowel preparation, and prior radiation therapy; and open surgery clustered with prior chemotherapy and comorbidities requiring chronic medication. BMI <25, alcohol use, and age ≤65 years showed contra-association with robotic surgery; elective status, prior radiation, and cancer resection showed contra-association with open surgery; and prior abdominal surgery was infrequently associated with laparoscopic technique. The largest standardized residuals, none reaching ±2 at a magnitude that would survive correction for multiple comparisons, were robotic surgery with elective procedures (residual = 2.7) and open surgery with cancer resection (residual = −2.6). The two plotted dimensions capture 100% of the total inertia (Dimension 1 = 71.1%, Dimension 2 = 28.9%) (Table 4).

Surgical technique and postoperative variables

Because 61.9% of expected cell counts were below 5, the likelihood-ratio chi-square is the appropriate test: G = 41.38, df = 26, p = 0.028 (Pearson χ² = 37.94, p = 0.061, for comparison). This is nominally significant at the conventional α = 0.05 threshold but does not survive Bonferroni correction (α = 0.01), a change from the original submission's blanket statement that this relationship was not statistically significant. Cramér's V = 0.22, a moderate effect (Table 3). Figure 5 shows laparoscopic surgery clustering with surgical site infection and intra-abdominal infection; open surgery clustering with blood product/vasopressor use, re-exploration, ICU admission, sepsis, anastomotic leak, and in-hospital death; and robotic surgery clustering with length of stay <6 days and return of bowel function <5 days. Open surgery showed contra-association with pneumonia and with length of stay <6 days; robotic surgery showed contra-association with re-exploration and sepsis. Standardized residuals ≥2 in magnitude were robotic surgery and return of bowel function <5 days (residual = 2.7); underuse of return of bowel function <5 days with open surgery (residual = −2.4); open surgery and ICU admission (residual = 2.3); open surgery and vasopressor/blood product use (residual = 2.2); absence of sepsis with robotic surgery (residual = −2.1); underuse of length of stay <6 days with open surgery (residual = −2.1); open surgery and in-hospital death (residual = 2.1); and open surgery and sepsis (residual = 2.0). Given the small absolute event counts for several of these outcomes (e.g., three deaths, three surgical site infections, seven anastomotic leaks total across the cohort), interpret these associations cautiously. The two plotted dimensions capture 100% of the total inertia (Dimension 1 = 86.4%, Dimension 2 = 13.6%) (Table 4).

Similarity and dissimilarity

In correspondence analysis, similarity and dissimilarity are assessed by the proximity of category points: categories located close together indicate a higher degree of similarity. In contrast, distant or oppositely directed points indicate dissimilarity. Similarity measures are essential for solving pattern recognition problems such as clustering, especially when performing multivariate analysis with multiple variables [9]. Among diagnoses, volvulus was the most dissimilar and distinctive category in the technique-diagnosis map, while sigmoid cancer was the least dissimilar. This is explained concretely by the underlying data: four of the five volvulus cases (80%) were managed laparoscopically and none robotically, a far more concentrated technique pattern than most other diagnoses show, placing the volvulus point well away from the origin and from the other diagnosis categories. Among preoperative variables, current smoking status was the most distinctive, and NSAID/anticoagulant use the least; among postoperative variables, in-hospital mortality was the most distinctive and pneumonia the least. Robotic technique was the most dissimilar/distinctive technique category in both the preoperative and postoperative analyses, and laparoscopic technique was the most dissimilar/distinctive in the technique-diagnosis analysis.

## Discussion

This study examined the association between surgical technique and diagnosis (primary aim) and, as secondary exploratory analyses, the associations between technique and preoperative/postoperative variables using correspondence analysis. While existing literature has compared the outcomes of open, laparoscopic, and robotic colorectal surgery, few studies have applied correspondence analysis to visualize multivariable relationships across preoperative, intraoperative, and postoperative variables simultaneously. This method offers a way to audit decision-making in a colorectal practice and to identify associations and contra-associations between technique and patient factors that might not be apparent from univariate comparisons alone.

The technique-diagnosis association -- the only analyses to remain significant after correction for multiple comparisons (G = 102.83, df = 28, p < 0.0001, Cramér's V = 0.51) -- showed laparoscopic surgery used most often for volvulus, appendiceal cancer, and colovaginal fistula; open surgery for sigmoid cancer, ostomy reversals, and peritoneal/gynecological cancer; and robotic surgery for ascending colon cancer, colovesicular fistula, and diverticulitis. Notable contra-associations included the near-total absence of open surgery for rectal cancer and descending colon cancer, and limited robotic use for colostomy creation and transverse colon cancer. These patterns are consistent with robotic technique being favored for complex pelvic dissection and reconstruction [10,11], and open technique remaining standard where extensive adhesiolysis or immediate exploration is required, as in ostomy reversal [12]. The laparoscopic preference for volvulus, appendiceal cancer, and colovaginal fistula -- the volvulus association being driven by four of five cases (see Results) -- more plausibly reflects this individual surgeon's preference and experience with these presentations than a generalizable technique indication, given the small cell counts involved.

In the technique-preoperative analysis (not statistically significant; χ² = 32.71, df = 26, p = 0.17), laparoscopic surgery was primarily associated with male patients and those on anticoagulants but was rarely performed in patients with prior abdominal surgery. Robotic surgery was associated with elective procedures, preoperative bowel preparation, and prior radiation therapy, while open surgery was more common in patients with comorbidities requiring long-term medications but was less frequently performed in elective cases. These findings align with previous research indicating that laparoscopic surgery is often avoided in patients with prior surgical history due to concerns regarding adhesions and technical difficulty [13-15]. Similarly, the strong association between robotic surgery and elective cases may reflect the increased operative time required for robotic procedures, making them less favorable in urgent or emergent settings [16]. Robotic surgery, associated with preoperative bowel preparation, reflects the rising approach where bowel preparation has been shown to reduce postoperative morbidity; a caveat is that bowel preparation with oral antibiotics used in combination reduces the risk of postoperative comorbidity [17]. The use of this combined bowel preparation is something we could not elicit in our data search. Robotic surgery's association with prior radiation therapy reflects standard oncologic management protocols, in which neoadjuvant therapy is frequently employed to reduce tumor burden before resection [18] in certain bowel cancers. This association also underscores the value of multidisciplinary collaboration in medical management and in selection of a surgical technique best suited to address radiation-related tissue changes encountered intraoperatively [19,20]. Additionally, through ERAS protocols, bowel prep reduces surgical site infections, accelerates bowel function recovery, and minimizes postoperative ileus, ensuring a smoother recovery and quicker resumption of adjuvant therapies [4,5].

Robotic surgery also showed contra-association with younger age, normal BMI, and alcohol use, plausibly reflecting that ascending colon cancer disproportionately performed using robotic technique in this practice is more common in older patients [21]. Notably, approximately 70% of patients were overweight or obese (Table 2). The high prevalence of elevated BMI within the study population may partly explain its frequent representation across the most commonly used surgical techniques (robotic surgery).

The preference for open surgery in patients with multiple comorbidities may not appear favorable, and concerns about poor outcomes associated with open procedures can be argued, as studies have shown that minimally invasive surgical techniques offer significant advantages over traditional open procedures, such as reduced blood loss, shorter hospital stays, fewer complications, and lower surgical costs [22]. However, the analysis suggests that this association can be explained by the fact that in this study, the comorbidities considered were diabetes mellitus, hypertension, thyroid disease, and immunodeficiencies. This spectrum of disease may make it difficult for wound healing, thus the initial requirement for a colostomy to avoid the breakdown of an anastomosis [23,24].

Postoperative variables varied by technique. Laparoscopic surgery was associated with higher rates of surgical site and intra-abdominal infections, a finding that contrasts with studies suggesting lower infection rates compared to open surgery [25]. However, total event counts were very low (three surgical site infections and nine intra-abdominal infections across the entire seven-year, 171-patient cohort), so this association is statistically detectable but not clinically meaningful in this practice. Open surgery's association with sepsis, ICU admission, vasopressor/blood product use, anastomotic leak, re-exploration, and in-hospital death is consistent with literature linking open technique to greater surgical trauma and morbidity [22], though here too the absolute numbers are small (e.g., three deaths total), and the global test for this relationship, while nominally significant (p = 0.028), did not survive correction for multiple comparisons. Robotic surgery's association with shorter length of stay and faster return of bowel function, and its contra-association with re-exploration and sepsis, are consistent with literature supporting robotic technique for enhanced precision and recovery [26,27], though again as an exploratory, uncorrected finding.

The distinctiveness of volvulus in the technique-diagnosis map reflects its unusually concentrated management pattern (four of five cases laparoscopic), suggesting either a strong, consistent surgeon preference for this presentation or a genuine clinical rationale (e.g., time-sensitivity favoring a technique the surgeon can perform quickly and reliably) that would be worth confirming prospectively. Sigmoid cancer's relative similarity to other diagnoses suggests a more standardized, technique-agnostic approach. The distinctiveness of current smoking status (present in only 29/171 patients, 17.0%) and its lack of association with any technique may reflect that smoking status per se does not drive technique choice in this practice, though we cannot confirm from this dataset whether structured smoking-cessation interventions were documented or delivered preoperatively [28]. Similarly, the distinctiveness of in-hospital mortality merits case-level review through standard Mortality & Morbidity conference processes [29] rather than further inference from the aggregate correspondence map alone, given the very small number of deaths (n = 3) involved.

Two additional practical considerations are relevant to interpreting these findings. First, robotic surgery's equipment, maintenance, and per-case costs are substantially higher than those of laparoscopic or open surgery; cost-effectiveness was outside the scope of this practice-pattern audit, and any decision to adopt a technique based on these findings should weigh that cost against the modest, uncorrected postoperative associations described above. Second, unlike robotic surgery, laparoscopic and open techniques do not require specialized capital equipment and remain feasible in resource-limited or remote settings; the technique distribution described here reflects a single, well-resourced community hospital and should not be read as a recommendation that would generalize to institutions without robotic platforms.

This study's findings can be applied directly to improve patient management and identify potential gaps in practice. Correspondence analysis helped identify which technique-diagnosis pairings were over- or under-represented relative to expectation, offering a template other surgeons or departments could apply to their own case mix. For example, the association between robotic surgery, shorter hospital stays, and faster bowel recovery may support its consideration in appropriately selected patients, although these findings were not statistically significant after correction.

Limitations

This is a retrospective, single-surgeon, single-institution observational study; the surgeon's individual training, preference, and evolving experience with each technique are not fully quantified in this dataset (see Methods), and the specific associations reported are unique to this practice. They cannot be generalized or externally validated without confirmation in a multi-surgeon, multi-institution cohort. Two of the five correspondence analyses performed -- technique versus preoperative variables and technique versus postoperative variables -- did not reach statistical significance at the Bonferroni-corrected threshold accounting for the five planned comparisons (α = 0.01); the technique-postoperative relationship was nominally significant only by the likelihood-ratio test (p = 0.028) and only before correction. Interpret the spatial associations from these two maps as exploratory and hypothesis-generating rather than confirmed findings. Several diagnostic categories are small (3-5 patients), which limits confidence in the corresponding technique associations and makes individual cells sensitive to a single additional or missing case. Several postoperative complications were rare events over the seven-year study period (e.g., three deaths, three surgical site infections), so associations involving them, while statistically detectable, are not necessarily clinically generalizable. Technique choice in real-world practice also depends on factors not captured as discrete variables here, including institutional equipment availability, individual patient anatomy and preference, and the surgeon's real-time intraoperative judgment. Finally, the substantially higher cost and limited availability of robotic techniques outside well-resourced centers further limit the generalizability of technique-specific recommendations drawn from this practice.

## Conclusions

Correspondence analysis is a useful method for examining relationships among surgical technique, diagnosis, and perioperative variables in colorectal surgery, simultaneously evaluating multiple categorical variables and identifying both associations and contra-associations. In this single surgeon's practice, the technique-diagnosis association was the only analysis to remain statistically significant after correction for multiple comparisons, with robotic surgery associated with ascending colon cancer and diverticulitis, and open surgery more frequently associated with ostomy reversal procedures. Associations between technique and preoperative or postoperative variables are presented as experimental and hypothesis-generating, as they did not survive correction for multiple comparisons (did not reach significance at all). The graphical output of correspondence analysis offers a straightforward way to visualize practice patterns and audit decision-making within a single dataset. Although these findings reflect one surgeon's experience at one institution and should not be generalized to other practices, the method itself is reproducible. It may help other surgeons evaluate their own past decision-making, identify practice patterns worth reviewing, and generate hypotheses for future prospective study.
